# Characterization of the complete chloroplast genome of *Ammopiptanthus mongolicus* (Leguminosae)

**DOI:** 10.1080/23802359.2021.1987174

**Published:** 2021-10-07

**Authors:** Jing Hu, Yan Peiying, Li Chang, Huiwen Zhang

**Affiliations:** State Key Laboratory Breeding Base of Desertification and Aeolian Sand Disaster Combating, Gansu Desert Control Research Institute, Lanzhou, China

**Keywords:** *Ammopiptanthus mongolicus*, chloroplast genome, *Ammopiptanthus*

## Abstract

*Ammopiptanthus mongolicus* is the only evergreen broadleaf shrub in the northwest desert of China, which can survive in long-term aridity and extremely cold environments. In this study, the complete chloroplast genome sequence of *A. mongolicus* was reported based on the Illumina NovaSeq Platform (Illumina, San Diego, CA). The chloroplast genome is 156,077 bp in length, containing a pair of inverted repeated (IR) regions (14,698 bp) that are separated by a large single copy (LSC) region of 88,025 bp, and a small single copy (SSC) region of 36,606 bp. Moreover, a total of 115 functional genes were annotated, including 81 mRNA, 30 tRNA genes, and 4 rRNA genes. Phylogenetic analysis based on 16 chloroplast genomics indicates that *A. mongolicus* is closely related to *A. nanus*.

*Ammopiptanthus mongolicus* is the only evergreen broadleaf shrub in the northwest desert of China, and is an endangered survivor from the Tethys in the Tertiary Period, which could grow well in long-term aridity and extremely cold environments (Liu et al. 2013). The habitats of *A. mongolicus* are marked by arid climate with an annual precipitation less than 200 mm and evaporation up to 3000 mm. Its natural distribution areas are also characterized as sandy or stony soil with high salinity, intense ultraviolet irradiation, and seasonally extreme temperature from about −30 °C in winter to more than 40 °C in summer. The extreme tolerance of this species to harsh environments makes it invaluable for exploring key stress-tolerant genes and mechanisms, especially those involved in cold and drought tolerance. Therefore, *A. mongolicus* has extremely important protective genetic value (Wu et al. 2014). However, genetic diversity of *A. mongolicus* has not yet been reported. Here we determined the complete sequence of the chloroplast genome of this plant compared the structures of genes and the genome of the *A. mongolicus* chloroplast with those of other plant species.

The fresh and young leaves of wild *A. mongolicus* collected from Minqin County (39°16´36.8^〃^N, 103°45´29.1^〃^E; elevation 1625 m) in Gansu Province, China. The voucher specimen and extracted DNA were deposited at Herbarium of Gansu Desert Control Research Institute under the number AM2020520-1 (Li Chang, alalei_2002@163.com). Total genomic DNA extraction and genome sequence assembling were conducted by Benagen Corporation (Wuhan, China). The locations of protein-coding genes were determined by comparing with the corresponding sequences of other species.

The complete genome of *A. mongolicus* is 156,077 bp in length with a typical quadripartite structure, containing a pair of inverted repeated (IR) regions (14,698 bp) that are separated by a large single copy (LSC) region of 88,025 bp, and a small single copy (SSC) region of 36,606 bp. The GC content of the whole genome was 36.87%. A total of 115 functional genes were annotated, including 81 mRNA, 30 tRNA genes, and 4 rRNA genes. The protein-coding genes, tRNA genes, and rRNA genes account for 70.43%, 26.09%, and 3.48% of all annotated genes, respectively.

The maximum-likelihood phylogenetic tree (ML tree) was generated based on the complete genome of *A. mongolicus* and other species of the family Leguminosae (Figure 1). Alignment was conducted using MAFFT (Katoh and Standley 2013), and the phylogenetic tree was built using RAxML (Stamatakis 2014). The results showed that *A. mongolicus* was closely related to *A. nanus*, supporting the view of *A. mongolicus* belongs to the genus *Ammopiptanthus* (Figure 1).

**Figure 1.**
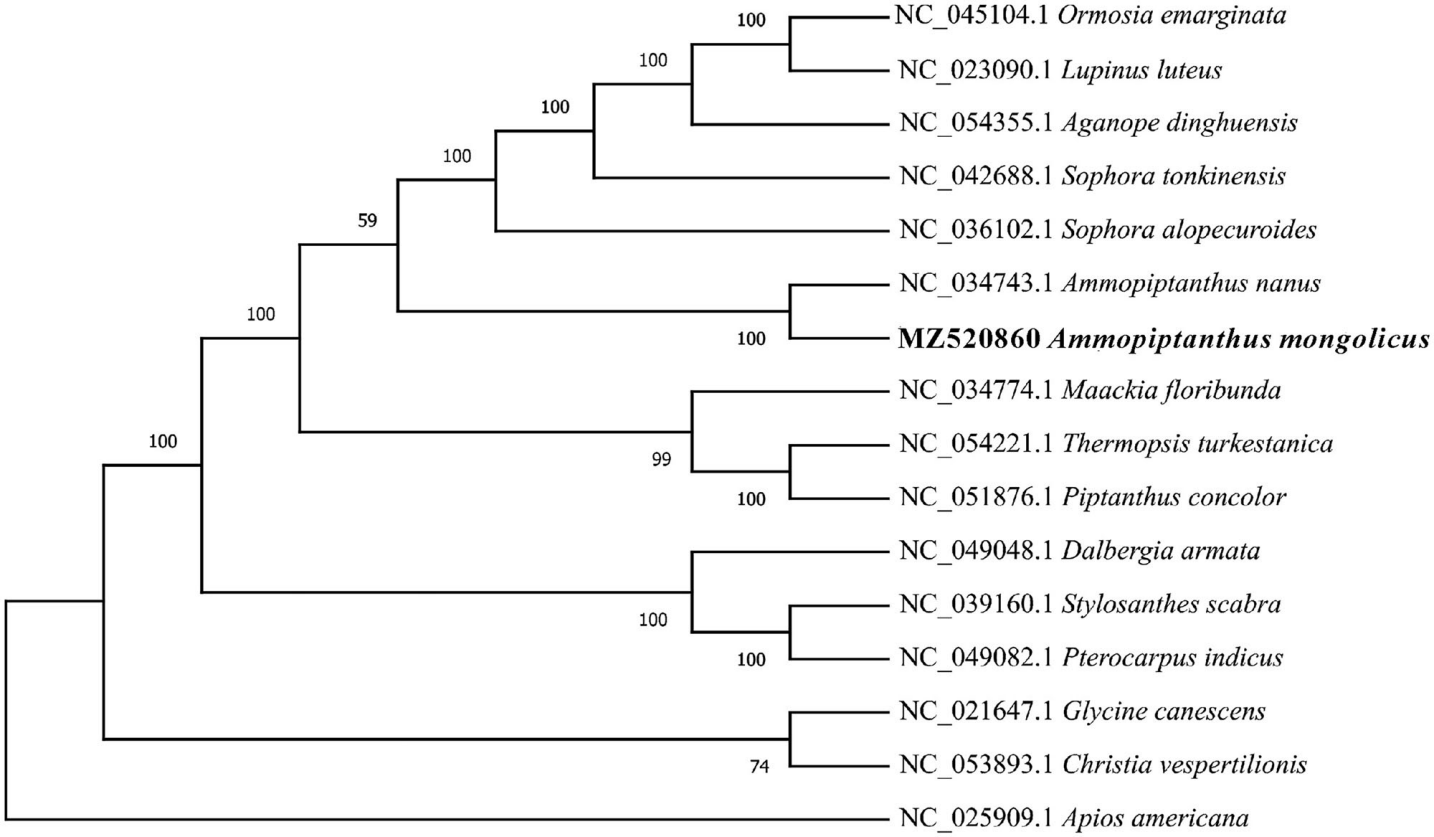
The ML tree based on 16 chloroplast genomes.

This study provided the characterization of the complete chloroplast genome structure of *A. mongolicus* and showed the phylogenetic relationships in Legume. All information about *A. mongolicus* chloroplast genome will provide a fundamental genetic reference for future efficient breeding of new trees and ornamental woody plants with improvement in abiotic stress tolerance.

## Data Availability

The genome sequence data that support the findings of this study are openly available in GenBank of NCBI at database (https://www.ncbi.nlm.nih.gov) under the accession number MZ520860. The associated Bio-Project, SRA, and Bio-Sample numbers are PRJNA743149, SRR15039806, and SAMN19999421, respectively.

## References

[CIT0001] Katoh K, Standley DM. 2013. MAFFT multiple sequence alignment software version 7: improvements in performance and usability. Mol Biol Evol. 30(4):772–780. 23329690 10.1093/molbev/mst010 PMC3603318

[CIT0002] Liu M, Shi J, Lu C. 2013. Identification of stress-responsive genes in *Ammopiptanthus mongolicus* using ESTs generated from cold- and drought-stressed seedlings. BMC Plant Biol. 13(1):88–114. 23734749 10.1186/1471-2229-13-88 PMC3679971

[CIT0003] Stamatakis A. 2014. RAxML version 8: a tool for phylogenetic analysis and post-analysis of large phylogenies. Bioinformatics. 30(9):1312–1313. 24451623 10.1093/bioinformatics/btu033 PMC3998144

[CIT0004] Wu Y, We W, Pang X, Wang X, Zhang H, Dong B, Xing Y, Li X, Wang M. 2014. Comparative transcriptome profiling of a desert evergreen shrub, *Ammopiptanthus mongolicus*, in response to drought and cold stresses. BMC Genomics. 15(1):1–16. 25108399 10.1186/1471-2164-15-671 PMC4143566

